# High-throughput glycomic analyses reveal unique oligosaccharide profiles of canine and feline milk samples

**DOI:** 10.1371/journal.pone.0243323

**Published:** 2020-12-03

**Authors:** David J. Wrigglesworth, Elisha Goonatilleke, Richard Haydock, Kevin R. Hughes, Carlito B. Lebrilla, Kelly S. Swanson, Paul Jones, Phillip Watson

**Affiliations:** 1 Waltham Petcare Science Institute, Waltham-on the-Wolds, Leicestershire, United Kingdom; 2 Department of Chemistry, University of California—Davis, Davis, California, United States of America; 3 Division of Nutritional Sciences, University of Illinois at Urbana-Champaign, Urbana, Illinois, United States of America; 4 Mars Incorporated, Mclean, Virginia, United States of America; Fisheries and Oceans Canada, CANADA

## Abstract

Oligosaccharides are important components of milk, serving as substrates for the intestinal microbiota, acting as antimicrobials that prevent pathogen colonization, and supporting the developing gastrointestinal immune system of neonates. Nutrient composition of canine and feline milk samples has been described previously, but little is known about the oligosaccharide content. Therefore, the objective of this study was to characterize canine and feline milk samples using a high-throughput glycomics approach. 23 dogs (9 Labrador retriever and 14 Labrador retriever x golden retriever crossbreed) and 6 domestic shorthair cats were recruited to the study. Milk samples were collected by manual expression at time points after parturition. Samples were collected across 2 phases per species, differentiated by maternal diet. Following extraction, oligosaccharide content was determined by liquid chromatography-mass spectrometry (LC-MS). In canine milk samples, 3 structures accounted for over 90% of all oligosaccharides detected across two diet groups. These were 3’-sialyllactose, 6’-sialyllactose, and 2’-fucosyllactose. In feline samples, a more diverse range of oligosaccharides was detected, with up to 16 structures present at relative abundance >1% of the total. Difucosyllactose-N-hexaose b, 3’-sialyllactose and lacto-N-neohexaose were all detected at abundances >10% in feline milk samples. Statistically significant differences (p<0.05) in oligosaccharide abundances were observed between collection time points and between diet groups within species. These data explore the oligosaccharide content of canine and feline maternal milk, representing an opportunity to generate a fundamental understanding of the nutritional needs of new-born puppies and kittens.

## Introduction

Maternal milk has been shaped by mammalian evolution to deliver the nutrition required for growth and development of neonates from birth until weaning. Consequently, it contains all essential macronutrients (water, vitamins, minerals, amino acids and fatty acids) needed to sustain early life. These nutrients are provided in maternal milk in a highly absorbable form that is appropriate for the stage of neonatal development. Maternal milk constituents, including macronutrients and microbes, vary widely across different species according to a complex interaction between the nutritional needs of the infant for growth and development, and the needs of the mother to preserve adequate nutritional status. These variations in the composition of milk and in the associated requirements of the neonate across species suggest adaptation of milk composition to the environmental niches, reproductive strategies, and nutrient and growth requirements of different mammalian infants [1].

On a dry matter basis, the third largest component in human milk is a group of lactose-derived human milk oligosaccharides (HMOs) [2, 3]. Infants are normally able to digest the lactose present in breast milk. However, the digestion and absorption of more complex carbohydrates, such as HMOs, requires a set of enzymes and intestinal membrane transporters that are not expressed by cells of the infant small intestine. Consequently, these HMOs are resistant to enzymatic hydrolysis in the upper gastrointestinal tract of the infant [4, 5] and reach the colon to function as prebiotics.

Specific data relating to the oligosaccharide (OS) composition of domestic canine and domestic feline milk are either scarce (canine) or non-existent in the literature at present (feline). Rostami *et al*. [6] reported the presence of 7 OS (plus lactose and lactose-3-sulphate) in milk samples from a panel of 7 dogs sampled at time points between day 1 and day 40 *post-partum*. Three OS were identified by mass/charge ratio as 2’-fucosyllactose (2’FL), 3’-sialyllactose (3’SL) and 6’-sialyllactose (6’SL). 3’SL was the more abundant OS in all breeds, with 6’SL being a tenth of the abundance and 2’FL abundance being variable between breeds. The only comparable feline study was reported by Senda *et al*. [7], describing the OS content of milk obtained from a single African lion (*Panthera leo*) and a single clouded leopard (*Neofelis nebulosa*), at 127 days and 1 day post-partum respectively. The samples were analysed by proton nuclear magnetic resonance and were found to contain 2’-fucosyllactose, A-tetrasaccharide and 3′-N-glycolylneuraminyllactose in the case of the African lion, and isoglobotriose and A-tetrasaccharide in the case of the clouded leopard. With these studies representing the only broadly relevant data reported in the literature, the full profile of OS in the milk of domestic cats and dogs is currently under-reported. Further exploration of the OS present in the milk of domestic dogs and cats across the course of lactation represents an important step in understanding the nutritional needs of new-born puppies and kittens, and the relationship between these prebiotic nutrients and the developing gut microbiome.

We hypothesised that it may be possible to manipulate the maternal diet in order to maximise the provision of certain OS in maternal milk. Meyer et al. [8] demonstrated that in human subjects, a high-fat diet decreased concentration of sialylated HMOs and a diet containing 60% calories from glucose resulted in a significant reduction in fucosylated HMOs compared to a diet containing 60% calories from galactose. The change in sialylated HMO concentrations in the milk was then shown to correlate with changes in infant gut microbiome diversity. The study reported in this paper here represents a first step in understanding the impact of maternal diet provision on milk OS production during lactation in dogs and cats.

## Methods

### Canine milk collections

Twenty-three dogs (9 Labrador retriever and 14 Labrador retriever x golden retriever crossbreed) were recruited to the study in 2 phases. These dogs belonged to the breeding population of a national assistance dog charity in the Unites States and were born and raised by volunteers within a home environment. A total of 12 dogs participated in phase 1 and 15 dogs in phase 2, with 4 dogs present in both cases. Mean (±SD) dog ages were 3.53 ± 1.32 years in phase 1 and 4.90 ± 0.92 in phase 2. Median (range) litter sizes were 4.8 (6–11) in phase 1 and 5.1 (6–11) in phase 2. Study phases were separated by a period of 4 months and differed according to maternal diet. During both sample collection phases, dogs were fed commercially prepared pet foods designed to be nutritionally complete and balanced for gestation and lactation. Phase 1 dogs were fed Eukanuba^®^ Premium Performance 30/20 throughout lactation and phase 2 dogs were fed Royal Canin^®^ Maxi Starter. Puppies were gradually weaned onto Eukanuba^®^ Large Breed Puppy from day 28 onwards. Milk samples were collected by manual expression directly into a 5ml cryovial, without the use of oxytocin, on days 2, 4, 6, 8, 10, 12, 17, 24, 31 and 38 *post-partum*, and were subsequently stored at -80°C until required for analysis.

### Feline milk collections

Six domestic shorthair cats were recruited to the study at the Iams Pet Health and Nutrition Center, US, and participated in 2 phases corresponding to consecutive mating cycles. Milk samples were collected during both mating cycles from 5 cats, with one cat excluded from the second phase due to poor sampling success in this individual. Data from this individual was retained in the analysis of phase 1. Mean (±SD) cat ages were 2.41 ± 1.52 years in phase 1 and 2.91 ± 1.52 in phase 2. During phase 1, milk samples were collected at 2-day intervals from days 8 to 34 post-partum. During phase 2, milk samples were collected on days 2, 4, 9, 11, 14, 16, 18, 21, 25, 32, and 39 post-partum. Differences in sampling schedule were to allow flexibility around other studies conducted at the same facility. Milk samples were obtained in the same manner as the canine collections and were subsequently stored at -80°C until required for analysis. Cats were maintained on Iams^®^ Kitten dry cat food (phase 1) or Royal Canin^®^ Feline Health Nutrition^™^ Mother & Babycat dry cat food (phase 2) at levels appropriate for gestation and lactation. Kittens were maintained on maternal milk as their sole source of nutrition throughout the study (weaning commenced from day 56).

The study was approved by the Institutional Animal Care and Use Committee (IACUC) of Canine Companions for Independence, US, in accordance with the Animal Welfare Act and Policy on Humane Care and Use of Laboratory Animals. The study was also approved by the Animal Welfare and Ethical Review Board of the Waltham Petcare Science Institute, UK.

**Fig 1:**
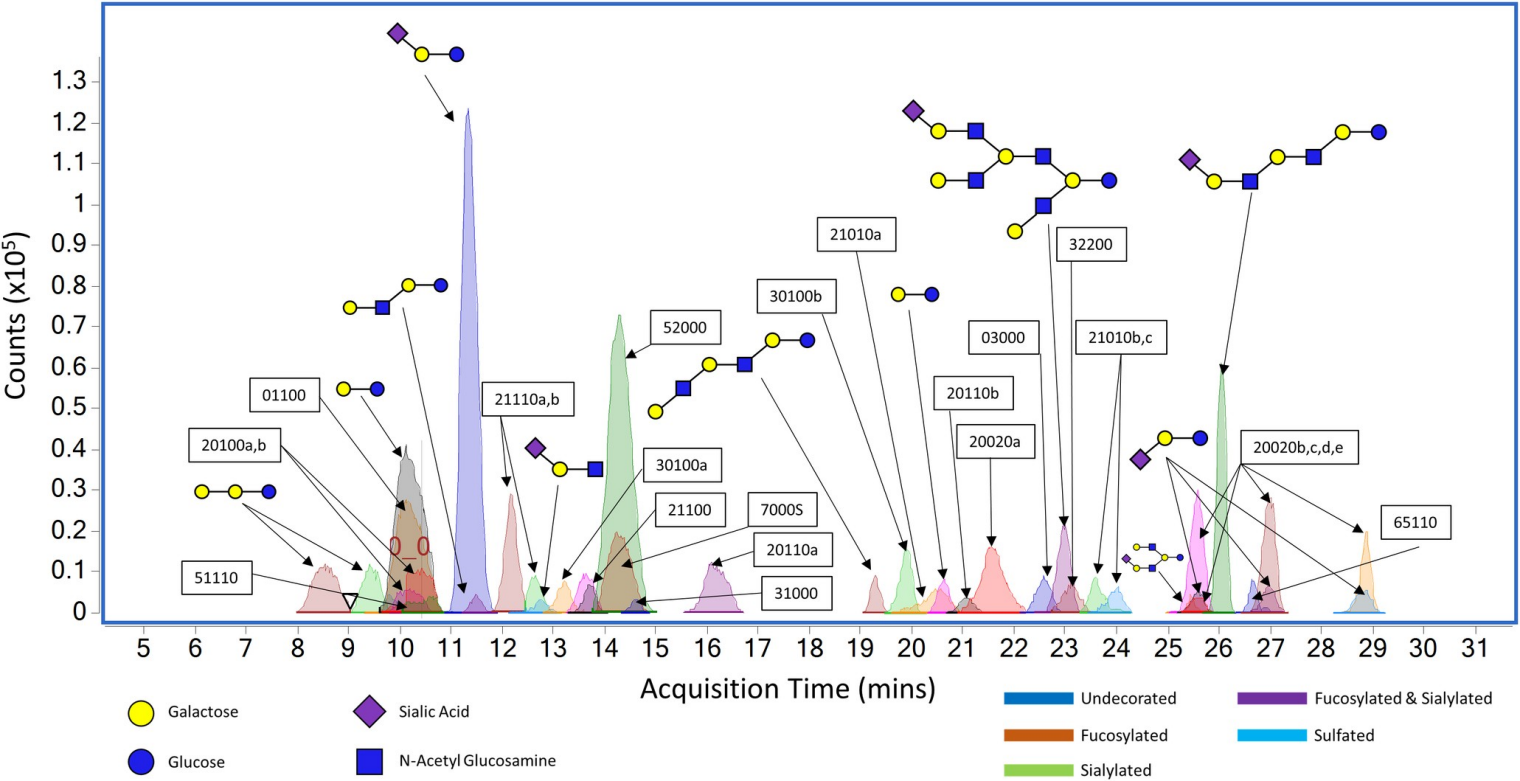
Example data obtained using HPLC Chip/TOF-MS to determine glycan signal in canine milk. All known compounds were extracted, overlaid and annotated, and were structurally identified using an in-house library. Each colour indicates a different glycan class–dark blue (undecorated neutrals), red (fucosylated), green (sialylated), purple (fucosylated and sialylated) and light blue (sulfated).

Milk OS analysis was carried out according to the extraction and LC-MS method reported by Wu *et al*. [9]. The protocol involves lipid separation via centrifugation, protein precipitation using ethanol, alditol reduction with sodium borohydride, and a final solid-phase extraction purification step using graphitized carbon cartridges. Samples were analysed using HPLC-Chip/TOF-MS and data filtered on Agilent Mass Hunter using an in-house library. Individual structural identification was matched against a previously developed HMO library using accurate mass and retention time (Fig 1). In the case of unnamed oligosaccharides, a 6 digit series was used to identify the number of hexose (Hex), N-acetylhexaoseamine (HexNAc), fucose (Fuc), N-acetylneuraminic acid (Neu5Ac, sialic acid), N-glycolylaminic acid (Neu5Gc), and sulfated residues respectively. Canine and feline samples were batch-assessed according to phase, with the exception of canine phase 2, which was assessed over two analysis runs.

### Data handling and statistics

Ion counts were normalised against a known reference pool of human milk OS, and these data were used in subsequent statistical analyses. Using these total ion counts, the relative abundance of each OS per animal per sample was calculated. Oligosaccharides with a reported ion count of 0 were assumed to be at the limit of detection and so were replaced with an estimate of the limit, calculated as 0.9*minimum of the total ion count and denoted as ‘censored’ observations. Two models were used to estimate mean normalised relative OS abundance per diet at each time point. In cases with no censoring (no values at the limit of detection), a linear mixed effects model was used. The response was set as abundance on a log10 scale, with sampling time *post-partum* and study phase as factors, their interaction as fixed effects, and animal per diet as a random effect. For OS with censored observations, a mixed effects extension of the Tobit model was used. This model incorporates information that the censored observations are lower (by an unspecified amount) than the value reported. For this model the same fixed and random effects structure as in the linear mixed effects model was used. For both of these models, the estimated mean OS abundances at each time point were estimated. The models were then refitted with time replaced by a time group indicator classifying days 2–14 as ‘Early’, days 16–28 as ‘Mid’ and days 30+ as ‘Late’. A series of hypothesis tests were performed comparing all sampling time groups to preceding time groups. For all hypothesis tests performed, the familywise error rate was controlled to achieve a 5% significance level. Subsets of the canine and feline OS data are presented here, representing the OS detected in both diet cohorts with average relative abundance greater than 5% over the course of lactation, and with significant time effects detected in at least one of the diet cohorts. The subsets consist of 6 OS from the feline data and 2 OS from the canine data. Since the canine data were dominated by a small number of OS, Lacto-N-neotetraose (LNnT) was also included, as this structure was detected in both diet cohorts and also demonstrated a significant time effect in both diet cohorts. All analyses were performed using R version 3.3.3 with the *lme4*, *multcomp* and *lmec* libraries.

## Results

**Fig 2:**
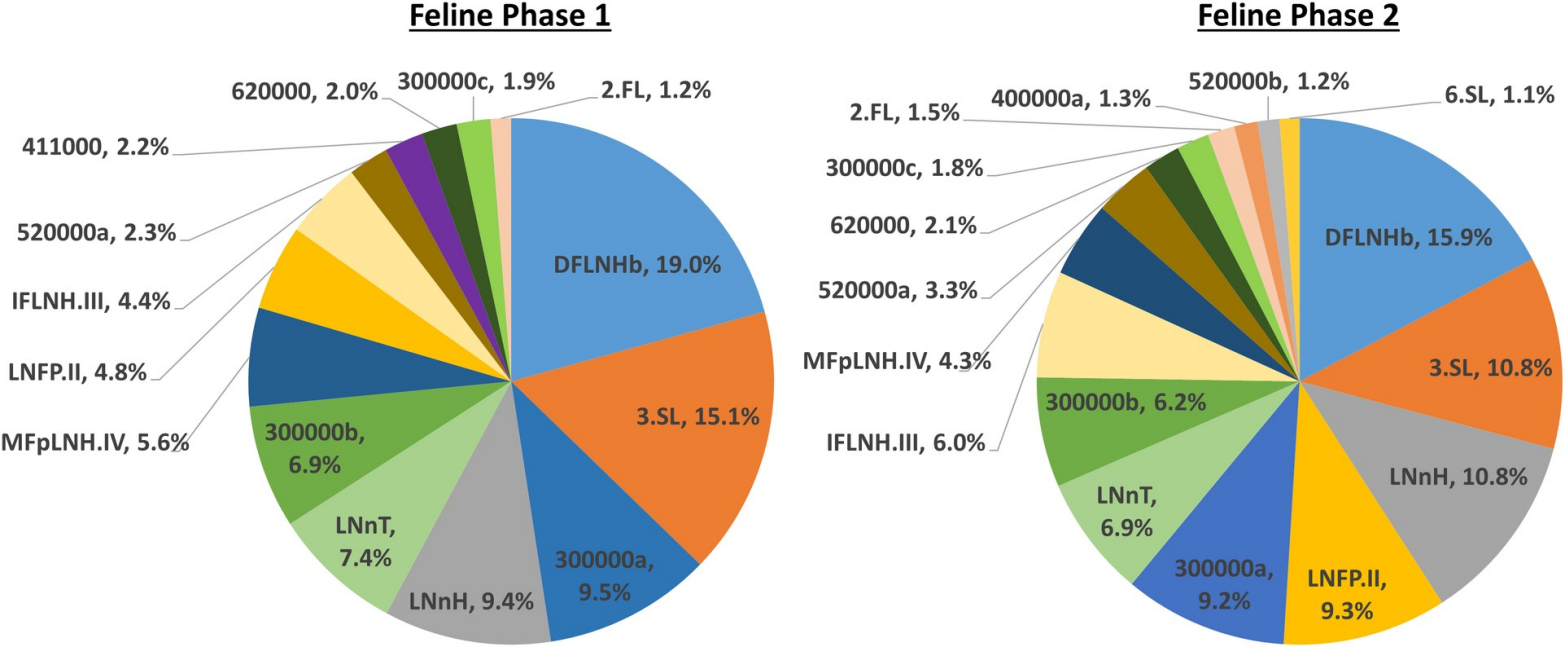
Mean normalised relative abundances of feline milk oligosaccharides. Proportion >1% for phase 1 and phase 2. Maternal diets: Phase 1 diet = Iams^®^ Kitten, Phase 2 diet = Royal Canin^®^ Feline Health Nutrition™ Mother & Babycat.

**Fig 3:**
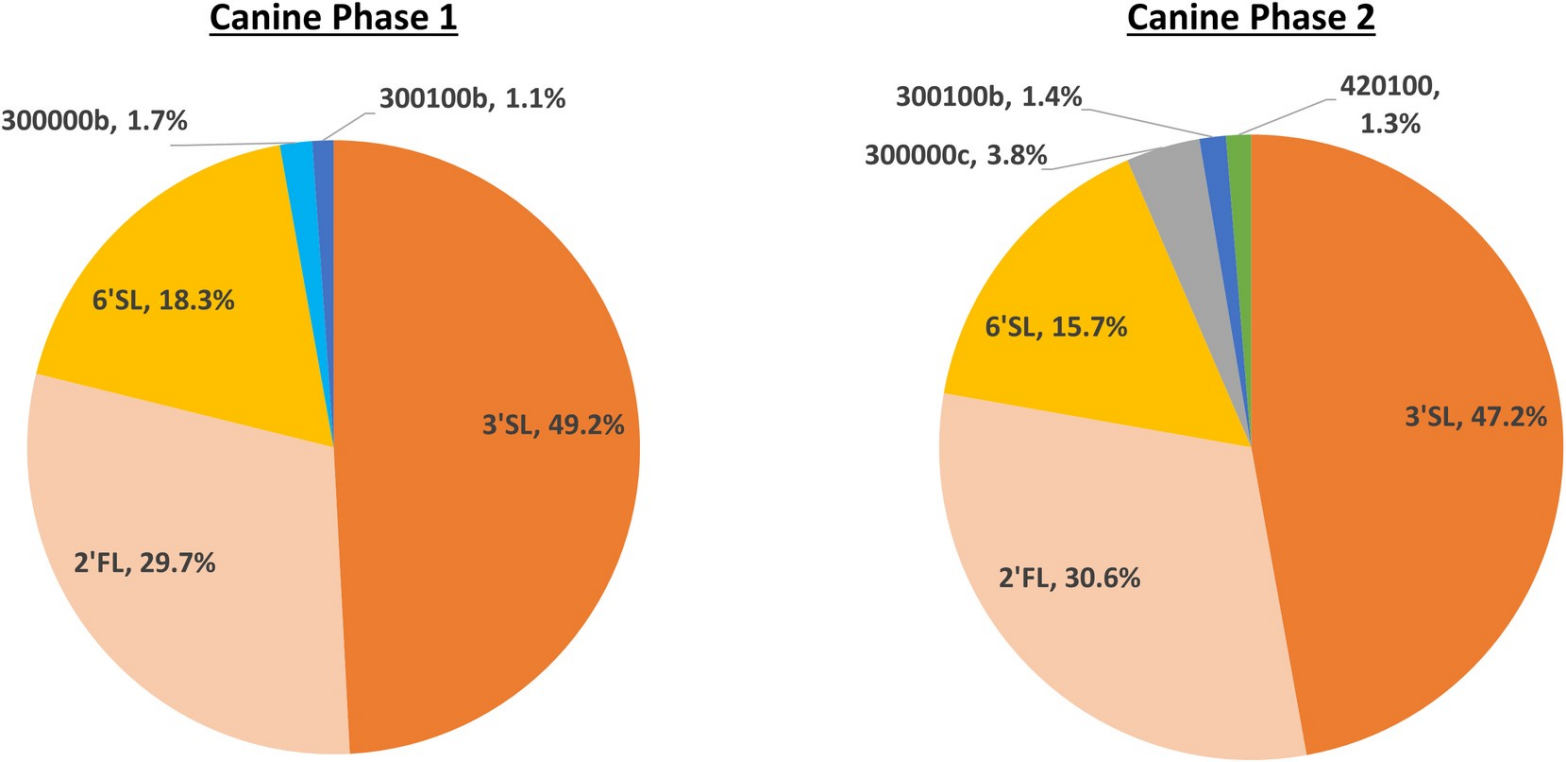
Mean normalised relative abundances of canine milk oligosaccharides. Proportion >1% for phase 1 and phase 2. Maternal diets: Phase 1 diet = Eukanuba^®^ Premium Performance, Phase 2 diet = Royal Canin^®^ Maxi Starter.

Fifty-five OS were identified in the canine milk samples, and 33 in the feline milk samples, with total ion count reported for each. All masses were able to be identified by accurate mass and retention time, with no requirement to utilise additional means (for example, tandem mass spectrometry MS/MS) to resolve individual peaks (S1 Table for feline data, S2 Table for canine data). Mean normalised relative abundances for milk OS with proportion >1% in each collection phase are presented for feline and canine data respectively (Figs 2 and 3). Canine milk predominately consisted of the OS 3’SL, 2’FL, and 6’SL, which together accounted for over 90% of the total. Feline milk displayed a comparatively even distribution of milk OS abundances, with both DFLNHb and 3’SL present at above 10% abundance in both phases, and between 14 and 16 structures present at abundances >1% of the total across lactation. Both canine and feline milk samples contained the sialic forms N-acetylneuraminic acid (Neu5Ac) and N-glycolyl neuraminic acid (Neu5Gc), indicating that, unlike humans, both species possess the hydroxylase required for this transformation.

**Fig 4:**
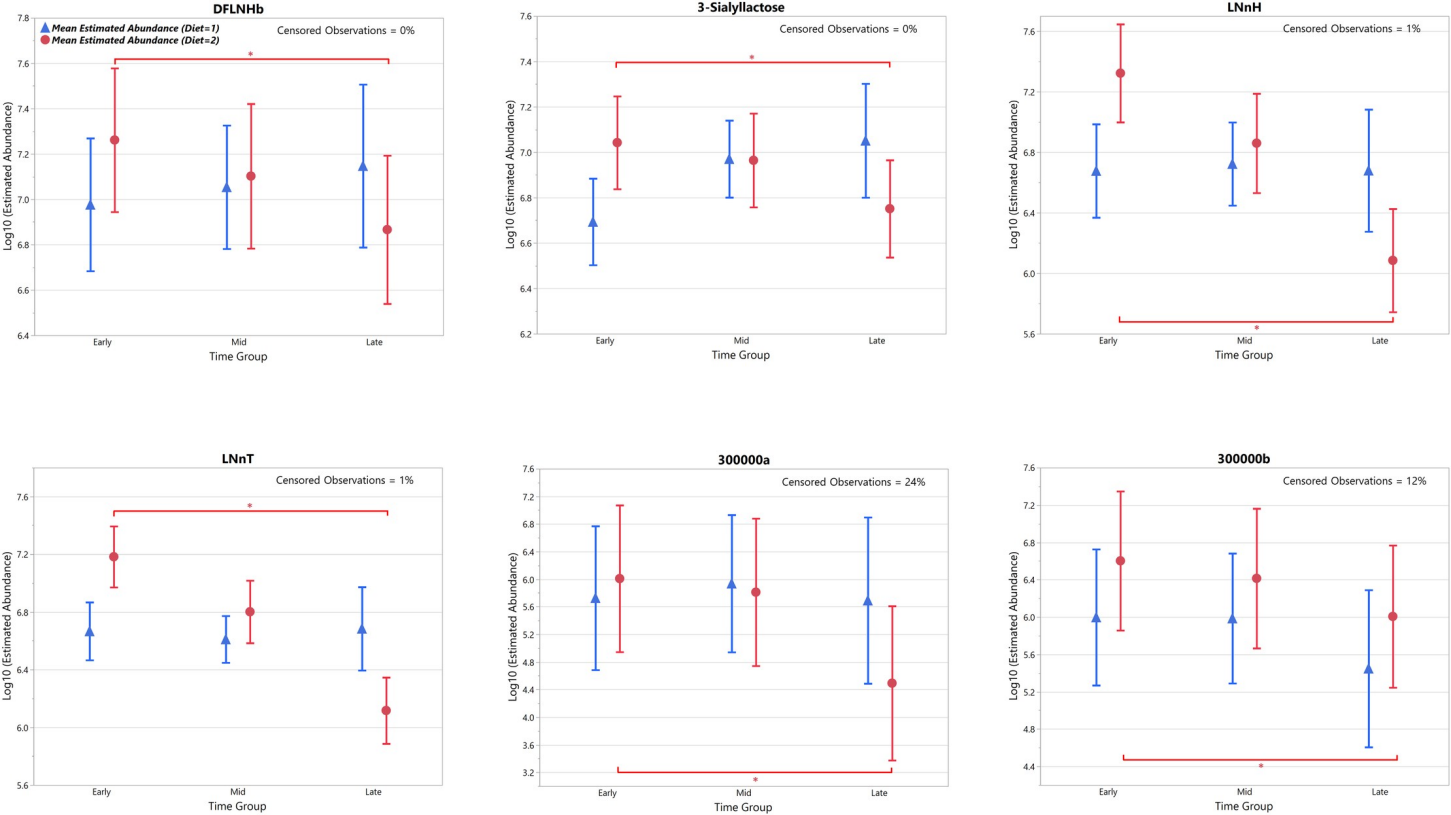
Estimated mean (95%CI) normalised abundance for the ‘Early’, ‘Mid’ and ‘Late’ time category groups of the feline milk oligosaccharides. Present at >5% of the total during the course of lactation. Asterisk bars indicate statistically significant differences within a diet group (p<0.05).

**Fig 5:**
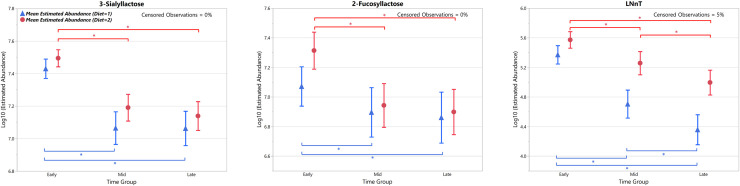
Estimated mean (95%CI) normalised abundance for the ‘Early’, ‘Mid’ and ‘Late’ time category groups of the canine milk oligosaccharides. Present at >5% of the total during the course of lactation, plus LNnT in addition. Asterisk bars indicate statistically significant difference within a diet group (p<0.05).

The estimated mean normalised abundance over the course of lactation for these OS, with 95% confidence intervals, are shown with means estimated for the time category groups (Figs 4 and 5). For the feline milk OS present at >5% of the total (Fig 4), 3’SL showed a significant increase in mean normalised abundance for cohort 1 between the Early vs. Mid and Early vs. Late time groups, and a significant decrease in mean normalised abundance for cohort 2 between the Early vs. Late collection time periods. Furthermore, a significant difference in 3’SL mean normalised abundance was detected between diets at both the Early and Late time periods, being higher in the phase 2 diet at the Early time period and higher in the phase 1 diet at the Late time period. The remaining OS (DFLNHb, 300000a, 300000b, LNnH and LNnT) all showed a significant decrease in mean normalised abundance for phase 2 between the Early vs. Late collection time periods. LNnH demonstrated a significant difference in mean normalised abundance between diets at both the Early and Late time periods, being higher in phase 2 at the Early time period and higher in phase 1 at the Late time period. 300000b demonstrated a significant difference in mean normalised abundance between diets at the Early time period, being higher in phase 2.

For the canine milk OS present at >5% of the total (Fig 5), both 3’SL and 2’FL showed a significant decrease in mean normalised abundance in both diet cohorts between the Early vs. Mid and Early vs. Late collection time periods. Mean normalised abundance of 2’FL was significantly higher in the diet 2 cohort compared to the diet 1 cohort at the Early collection time period. Additionally, LNnT showed significant decreases in mean normalised abundance for both diet phases between all collection time periods, and levels of LNnT were significantly higher in diet phase 2 compared to diet phase 1 at all collection time periods.

## Discussion

Milk from 23 dogs and 6 cats was successfully sampled over the course of lactation, for two collection phases during which both the dogs and cats received different diets. These samples were then analysed to provide data on the abundance of 55 canine and 33 feline milk OS structures over this timeframe, and within-species contrasts between abundance in defined time categories were performed.

The most notable observation is the difference in the relative distribution of feline milk OS when compared to canine milk. Canine milk is characterised by the dominance of a limited number of OS, with 3’SL, 6’SL and 2’FL accounting for over 90% of all structures detected. The distribution of feline milk OS is broader by comparison, with the 10 most prevalent OS accounting for approximately 70% of the total, highlighting the fact that different species may have different requirements for the provision of milk OS [1]. In general, it is useful to draw comparisons between the results of this study and the oligosaccharide profiles in human and bovine milk, which have been extensively researched. The abundance of fucosylated, sialylated, and nonfucosylated neutral oligosaccharides in human breast milk has been reported as 35–50%, 12–14%, and 42–55% respectively [10]. This is notably similar to the composition of feline milk reported in the current study, with higher proportions of fucosylated and non-fucosylated neutral structures, and a relatively low level of sialylated structures. In the canine milk samples reported here, the reverse is apparent, with very high abundance of sialylated structures being observed. In this respect, canine milk is more similar to bovine milk, which has been reported to contain high levels of 3’-sialyllactose and other sialylated structures [11]. Sialic acid is an essential nutrient in the development of the neonatal brain, being used in the formation of brain gangliosides and sialylated glycoproteins that play a role in cell-cell interactions, neuronal outgrowth, synaptic connections and memory formation [12]. A diet rich in sialic acid has been shown to enhance learning and memory development in rodents [13, 14] and piglets [15]. Human studies have shown that formula milk contains lower levels (<25%) of sialic acid compared to mature human breast milk [16], and that breast-fed preterm infants demonstrate higher developmental scores at 18 months of age [17] and have higher intelligence quotients at age 7 [18]. There is also compelling evidence to suggest that sialylated milk OS selectively promote the adhesion of beneficial bacteria, thereby conferring a competitive advantage on these species, and also play a role in modulating inflammation and immunity, both *in vitro* and in *vivo* [19, 20]. Taken together, this evidence suggests that the high level of sialic acid found in maternal milk, principally as sialylated OS, plays an important and diverse role in neonatal development, and it therefore seems unlikely that an OS that provides such a material benefit is not provided in sufficient quantity to the kitten through the maternal milk. Furthermore, variation in sialylated oligosaccharide content has been described previously in a range of domestic animals [21, 22]. Consequently, lower levels of sialylated OS provision in domestic shorthair cats may be related to a lower neonatal requirement, to higher levels of neonatal *de novo* synthesis, or to some functional redundancy with respect to non-sialylated OS.

Comparing the canine results obtained in this study with those reported elsewhere, they are generally consistent with those of Rostami *et al*. [6], in which 3’SL, 6’SL and 2’FL were the predominant OS detected in a panel of dogs consisting of more than one breed, with 3’SL being the most abundant. An apparent breed effect on milk OS profile was also noted, with 2’FL being more highly represented in the miniature schnauzer breed and a tetrasaccharide being present only in the Alaskan husky breed, and although a robust statistical analysis of breed specific differences in milk OS profile was not made, such breed differences have been previously reported in bovine [23] and equine [24] studies. Similarly, the current study was not powered to detect breed differences in OS profile, but levels of LNnT were observed to be notably higher at the ‘Late’ time point in purebred Labrador Retrievers compared to Labrador x Golden retriever crosses. Conversely, levels of 300100b were higher at the ‘Late’ time point in Labrador x Golden retriever crosses compared to purebred Labrador Retrievers. Hence, the potential for these breed differences to exist should be noted, and care should be exercised when comparing results between breeds.

Although the data from this study focussed on OS structures with relative abundance >5%, it should not be assumed that OS present at lower abundances are biologically inconsequential. Their presence in the pool of maternal milk OS suggests that they perform some beneficial function, since there has been an evolutionary pressure for them to be preserved. However, given that highly abundant OS have been identified within the milk of both dogs and cats, including statistically significant time effects over the course of lactation, it seems appropriate to prioritise further investigation of these structures in the first instance. It is also notable that a consistent trend towards lower relative abundance across time is observed for the 3 most prevalent canine milk OS, whereas feline milk OS are observed to be more variable. This may relate to the lower sample size in the feline data reported here, and a similar trend should not be discounted in cats compared to dogs.

The study design included the opportunity to investigate the effect of different maternal diets on milk OS profile. Ideally, in order to establish whether such differences exist, diets would be chosen to be as divergent as possible, either in terms of macronutrient profile, digestibility, or some other characteristic related to overall diet quality. However, these choices were limited by the need to ensure that the puppies and kittens were not nutritionally disadvantaged, that they received the nutrition from maternal milk required to grow and thrive, and that the dams were not at risk of undernutrition during lactation. Hence, to purposefully select a poor quality maternal diet in order to investigate the effect of this on the oligosaccharide profile of the maternal milk would be ethically questionable. Consequently, the diets were chosen from a limited number of possible options, namely those that were specifically designed to provide maternal nutrition during lactation, and the differences in macronutrient profile were therefore relatively modest, both being high in protein and fat content. Despite this, a number of statistically significant differences in the quantity of specific OS was observed during the study. In particular, levels of LNnT and 2’FL were higher in some or all aspects of the phase 2 samples compared to the phase 1 samples. Specifically, 2’FL was significantly higher during the Early phase of lactation and LNnT was significantly higher at all time groups (Early, Mid, and Late). Both LNnT and 2’FL have been shown in human studies to promote changes in the microbiome towards increased levels of *Bifidobacterium* [25]. This genus has been associated with a healthy human gut microbiota, with low levels reported in obese and diabetic individuals [26, 27] and in patients suffering from irritable bowel syndrome or inflammatory bowel disease [28, 29]. The potential for some maternal diet components to promote the growth of *Bifidobacteria* in the neonate warrants further investigation, since it is not clear what aspect of the diets in this study was driving the differences observed.

Our results demonstrate that the milk produced by domestic dogs and cats is unique in terms of its OS profile, although similarities were noted with bovine and human milk oligosaccharide content respectively, and that further investigation may be required in order to gain a wider understanding of breed-specific OS profiles within species. These findings represent opportunities to support the provision of prebiotic milk oligosaccharides to puppies and kittens during early life, in order to promote optimal establishment of the initial colonising gut microbiota.

## Supporting information

S1 Table Tabulated abundances for all oligosaccharides detected in feline milk samples. Counts are notated according to diet consumed and post-partum day of milk sample collection.
(XLSX)

S2 Table Tabulated abundances for all oligosaccharides detected in canine milk samples. Counts are notated according to diet consumed and post-partum day of milk sample collection.
(XLSX)
